# Supplementary material for: Shedding Light on Viral Shedding: Novel Insights into Nuclear Assembly, Cytoplasmic Transformation and Extracellular Vesicle Release of the BK Virus
Source: Int J Mol Sci. 2024 Aug 22;25(16):9130. doi: 10.3390/ijms25169130 (PMC11354704; doi:10.3390/ijms25169130)
Supplement: Supplementary file 1 [file ijms-25-09130-s001.zip › ijms-3091366-supplementary.pdf]

Supplementary to the manuscript

Shedding light on viral shedding: Novel Insights into Nuclear Assembly, Cytoplasmic Transformation and Extracellular Vesicle Release of the BK Virus

Daniela Gerges [1,†], Karim Abd El-Ghany [1,2,†], Zsofia Hevesi [2], Monika Aiad [1], Haris Omic [1], Clemens Baumgartner [1,3], Wolfgang Winnicki [1], Michael Eder [1], Alice Schmidt [1], Farsad Eskandary [1,$,*] and Ludwig Wagner [1,§]

Data set S1

Amino acid sequence of BK virus Vp1 protein used for rabbit Immunization

MAPTKRKGECPGAAPKKPKEPVQVPKLLIKGGVEVLEVKTGVDAITEVECFLNPEMGDPDDHLRGYSQH
LTAENAFDSDSPDKKMLPCYSTARIPLPNLNEDLTCGNLLMWEAVTVKTEVIGITSMLNLHAGSQKVHE
NGGGKPVQGSNFHFFAVGGDPLEMQGVLMNYRTKYPQGTITPKNPTAQSQVMNTDHKAYLDKNNA
YPVECWIPDPSKNENTRYFGTYTGGENVPPVLHVTNTATTVLLDEQGVGPLCKADSLYVSAADICGLFT
NSSGTQQWRGLARYFKIRLRKRSVKNPYPISFLLSDLINRRTQKVDGQPMYGMESQVEEVRVFDGTEQL
PGDPDMIRYIDRQGQLQTKMV

Coding sequence in pET-30a bacterial protein expression plasmid encoding BK-Vp1 genotype I

ATGGCACCGACCAAACGTAAAGGTGAATGTCCGGGTGCAGCACCGAAAAAGCCGAAAGAACCGG
TGCAGGTTCCGAAACTGCTGATTAAAGGTGGTGTTGAAGTTCTGGAAGTTAAAACCGGTGTTGATG
CAATTACCGAAGTTGAATGTTTTCTGAATCCGGAAATGGGTGATCCGGATGACCATCTGCGTGGTTA
TAGCCAGCATCTGACCGCAGAAAATGCCTTTGATAGCGATAGTCCGGATAAAAAGATGCTGCCGTG
TTATAGCACCGCACGTATTCCGCTGCCGAATCTGAATGAAGATCTGACATGTGGTAATCTGCTGATG
TGGGAAGCAGTTACCGTGAAAACCGAAGTTATTGGTATTACGAGCATGCTGAATCTGCATGCCGGT
AGCCAGAAAGTTCATGAAAACGGTGGTGGTAAACCGGTTCAGGGTAGCAATTTTCATTTCTTTGCA
GTTGGTGGTGATCCGCTGGAAATGCAGGGTGTTCTGATGAATTATCGTACCAAATATCCGCAGGGT
ACAATTACCCCGAAAAATCCGACCGCACAGAGCCAGGTTATGAATACCGATCATAAAGCATACCTG
GACAAAAATAACGCCTATCCGGTTGAATGCTGGATTCCGGATCCGAGCAAAAATGAAAACACCCG
TTATTTTGGCACCTATACCGGTGGTGAAAATGTTCCGCCTGTTCTGCATGTTACCAATACCGCAACC
ACCGTTCTGCTGGATGAACAAGGTGTTGGTCCGCTGTGTAAAGCAGATAGCCTGTATGTTAGCGCA
GCAGATATTTGTGGTCTGTTTACCAATAGCAGCGGCACCCAGCAGTGGCGTGGTCTGGCACGTTAT
TTCAAAATTCGTCTGCGTAAACGCAGCGTGAAAAACCCGTATCCGATTAGCTTTCTGCTGAGCGATC
TGATTAATCGTCGTACCCAGAAAGTGGATGGTCAGCCGATGTATGGTATGGAAAGCCAGGTTGAAG
AGGTTCGTGTTTTTGATGGTACAGAACAGCTGCCTGGCGATCCGGATATGATTCGTTATATTGATCG
TCAGGGTCAGCTGCAGACCAAAATGGTTTAA

Data set S2

Amino acid sequence of BK virus Vp1 protein used for generating recombinant BK virus Vp1 protein genotype I

MAPTKRKGECPGAAPKKPKEPVQVPKLLIKGGVEVLEVKTGLDAITEVECFLNPEMGDPDENLRGFSLKL
SAENNFSSDSPERKMLPCYSTARIPLPNLNEDLTCGNLLMWEAVTVQTEVIGITSMLNLHAGSQKVHEH
GGGKPIQGSNFHFFAVGGDPLEMQGVLMNYRTKYPEGTITPKNPTAQSQVMNTDHKAYLDKNNAYP
VECWIPDPSRNENTRYFGTFTGGENVPPVLHVTNTATTVLLDEQGVGPLCKADSLYVSAADICGLFTNSS
GTQQWRGLARYFKIRLRKRSVKNPYPISFLLSDLINRRTQRVDGQPMYGMESQVEEVRVFDGTEKLPGD
PDMIRYIDKQGQLQTKML

Coding sequence in pEXP1-DEST bacterial protein expression plasmid encoding BK-Vp1 genotype I

ATGGCCCCAACCAAAAGAAAAGGAGAGTGTCCAGGGGCAGCTCCCAAAAAGCCAAAGGAACCCG
TGCAAGTGCCAAAACTGCTAATAAAAGGAGGAGTAGAAGTTCTAGAAGTTAAAACTGGGCTAGAT
GCTATAACAGAGGTAGAATGCTTCCTAAACCCAGAAATGGGGGATCCAGATGAAAACCTTAGGGG
CTTTAGTCTAAAGCTAAGTGCTGAAAATAACTTTAGCAGTGATAGCCCCGAAAGAAAAATGCTTCC
CTGTTACAGCACAGCAAGAATTCCCCTCCCCAATTTAAATGAGGACCTAACCTGTGGAAATCTACT
GATGTGGGAGGCTGTAACAGTACAAACAGAGGTCATTGGAATAACTAGCATGCTTAACCTTCATGC
AGGGTCACAAAAAGTGCATGAGCATGGTGGAGGTAAACCTATTCAAGGCAGTAATTTCCACTTTTT
TGCTGTTGGTGGAGACCCCTTGGAAATGCAGGGAGTGCTAATGAATTACAGGACAAAGTACCCAG
AAGGTACTATAACCCCAAAAAACCCAACAGCCCAGTCCCAAGTAATGAATACTGACCATAAGGCCT
ATTTGGACAAAAACAATGCTTATCCAGTTGAGTGCTGGATTCCTGATCCCAGTAGAAATGAAAATA
CTAGGTATTTTGGGACTTTCACAGGAGGGGAAAATGTTCCCCCAGTACTTCATGTGACCAACACAG
CTACCACAGTGTTGCTAGATGAACAGGGTGTGGGGCCTCTTTGTAAAGCTGATAGCCTGTATGTTTC
AGCTGCTGATATTTGTGGCCTGTTTACTAACAGCTCTGGAACACAACAGTGGAGAGGCCTTGCAAG
ATATTTTAAGATTCGCCTGAGAAAAAGATCTGTAAAAAATCCTTACCCAATTTCCTTTTTGCTAAGTG
ACCTTATAAACAGGAGAACCCAGAGAGTGGATGGGCAGCCTATGTATGGTATGGAATCCCAGGTA
GAAGAGGTTAGGGTGTTTGATGGCACAGAAAAACTTCCAGGGGACCCAGATATGATAAGATATATT
GACAAACAAGGACAATTGCAAACCAAAATGCTTTAA

Supplementary Figure 1

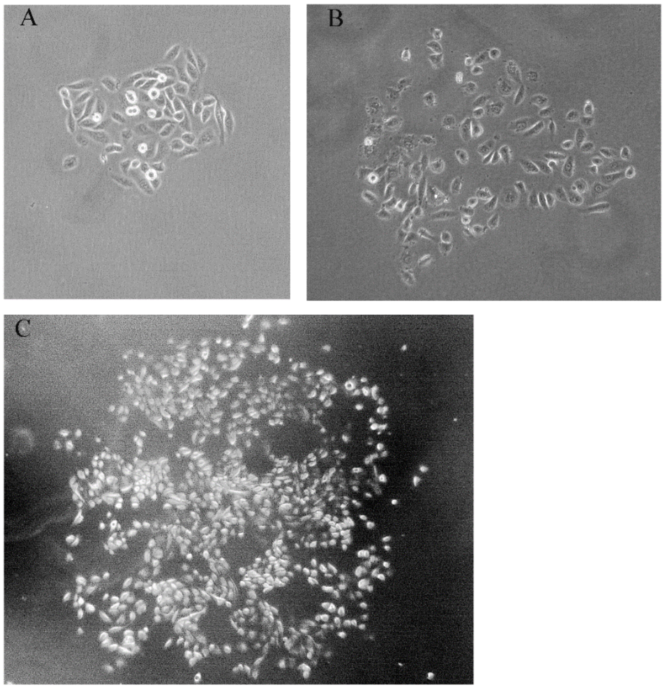

Supplementary Figure 1. Urinary renal epithelial cell clones in small tissue culture plates (3.5cm) multiplied into colonies of up to approximately 30 cells (A) within 6-7 days. These colonies expanded, with some cells migrating to the periphery apart from the original clone (B, day 9), eventually reaching expansive growth and forming multilayers in some areas (C, day 13). Data were taken from patient #4.

Supplementary Figure 2

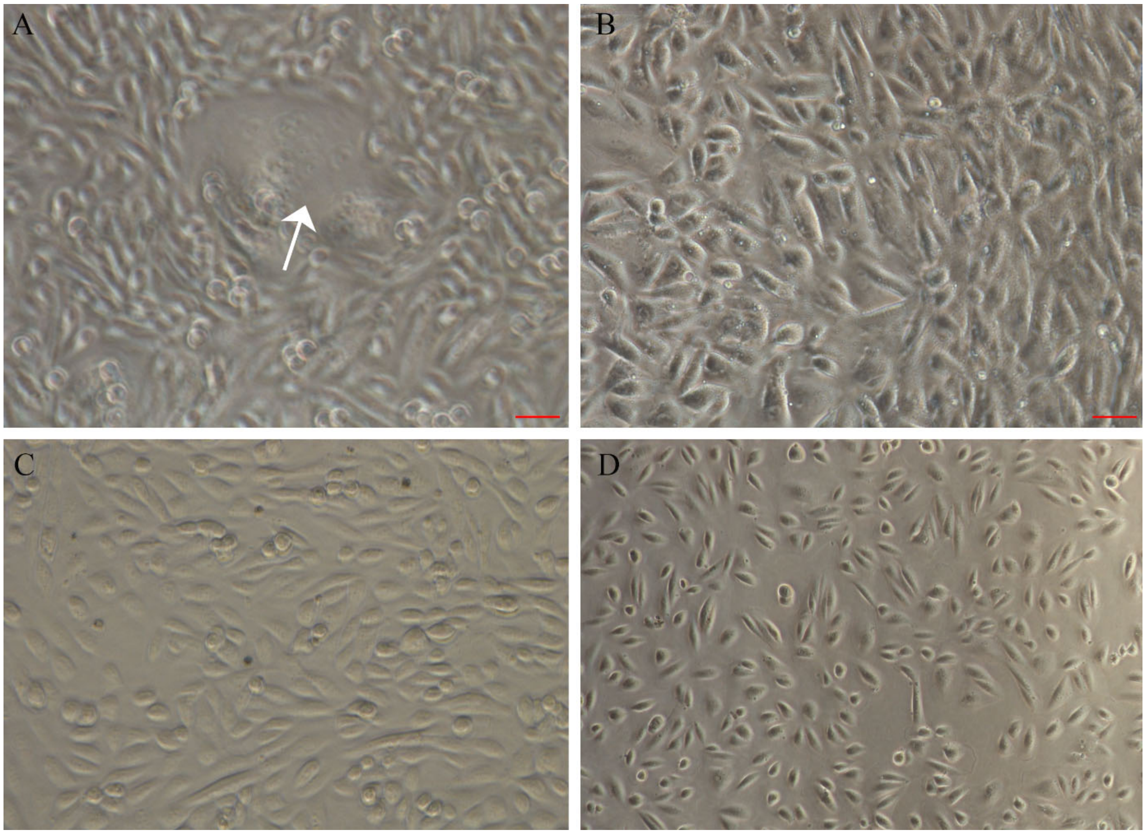

Supplementary Figure 2. Confluent cells layer of patient #4 and polyploidy cell as indicated by arrow (A), confluent cell layer of cell line from patient #6 (B), and patient #7 (C). Patient #5 is depicted in semi-confluent stage (D).
